# The time spent sitting does not always mean a low level of physical activity

**DOI:** 10.1186/s12889-020-8396-3

**Published:** 2020-03-12

**Authors:** Ewelina Matusiak-Wieczorek, Anna Lipert, Ewa Kochan, Anna Jegier

**Affiliations:** 1 Sports Medicine Institute, Social and Preventive Medicine Department, Medical University of Lodz, Pomorska 251, 92-213 Lodz, Poland; 2 Pharmaceutical Biotechnology Institute, Pharmaceutical Biology and Biotechnology Department, Medical University of Lodz, Lodz, Poland

**Keywords:** Sitting time, Sedentary behaviour, Physical activity level, IPAQ

## Abstract

**Background:**

The problem of spending most of the day in a sitting position concerns all people, regardless of their age. Unfortunately, this trend is more and more often observed among young people.

The aim of the study was to assess self-reported physical activity and time spent sitting among students of different fields of health related faculty.

**Methods:**

The study group included 216 students (22.3 ± 1.8 years of age) of the Medical University of Lodz: physiotherapy students (*n* = 101), pharmacy students (*n* = 73), and dietetics students (*n* = 42). The time spent sitting and physical activity level were assessed based on the International Physical Activity Questionnaire-long version.

**Results:**

The time spent sitting among health related faculty students was on average more than 46 h a week (2781.8 ± 1238.5 MET-minutes/week). Regarding all the students the pharmacy students spent most time sitting (3086.0 ± 1032.1 MET-minutes/week), while the dietetics students spent the least (2215.7 ± 1230.1 MET-minutes/week). Taking into account the physical activity level almost 65% of all the students were in a high category (mainly physiotherapy students). Only 1.4% of all the surveyed students were classified as the low physical activity category. Statistical analysis showed no significant differences (*P* = 0.6880) between the time spent sitting and level of physical activity among all students.

**Conclusions:**

Students of medical universities spend too much hours on sitting, mostly 5–8 h a day. Despite this, they undertake various activities due to which their level of physical activity is moderate or even high. Therefore, it cannot be unequivocally stated that there is a relationship between the time spent sitting and physical activity level.

## Background

Many studies report that people are inactive most of the day and spend their time mainly sitting [1–6]. Such a sedentary lifestyle is associated with a greater risk of cardiovascular, metabolic and cancer diseases, which can lead to an increased overall mortality [1–3, 7].

The problem of the decline in the level of physical activity while increasing the time spent sitting is global and affects people of all ages [1, 8–11]. Research studies highlight that this situation is particularly characteristic for young people [1, 12–14]. One of the mentioned groups is university youth. University studies are the last stage in young people’s lives at which they have a chance of acquiring proper knowledge and healthy habits before beginning an independent adult life [15]. It is extremely important to develop such an attitude among students of medical universities. Apart from all the health benefits they themselves may get from physical activity, in their future work they will be much better prepared to promote a healthy lifestyle among their patients [16–18]. Further research will allow for a thorough examination of the current physical activity level and the sedentary behaviour among this group of individuals. To the best of our knowledge this study is the first one to investigate the amount of time during waking hours students of health related faculty spend sitting and then to compare the obtained results with their physical activity level.

The aim of the study was to assess sitting and activity among students of different fields of health related faculty, based on the long version of the International Physical Activity Questionnaire (IPAQ-LV). In the following study, we hypothesized that sitting is not associated with physical activity.

## Methods

The study was conducted in 2018, among 216 students (44 males and 172 females; 22.3 ± 1.8 years of age) of the Medical University of Lodz. The study included physiotherapy students - 101 (46.8%), pharmacy students - 73 (33.8%), and dietetics students - 42 (19.4%). Students who studied only in one of the listed fields of health related faculty were included into the study. The study excluded those who studied simultaneously at more than one of the mentioned fields.

Participation in the study was voluntary. The students were informed about the aims and methods of the study and then they provided written consents for participation in it. Afterwards, the students were asked to complete the long version of IPAQ. They answered questions by indicating the number of days per week and time (in hours and minutes) they spent walking, being involved in moderate, vigorous intensity activities and sitting. The questionnaire referred to the physical activity of the last seven days. Each single activity had to take at least 10 min to be included. The students completed the IPAQ-LV in the presence of an interviewer, who explained any doubts to the respondents. The confidentiality of the data and the anonymity of the participants were ensured throughout the whole study.

When the questionnaires were collected, the data contained therein was processed in accordance with the scoring protocol available at www.ipaq.ki.se. Based on that information about the time spent sitting during the whole week and average day was obtained and all the participants were classified into the appropriate physical activity levels: low, moderate, high.

### Statistical analysis

The data in this study was presented as mean values with standard errors.

The relationship between the time spent sitting and the level of physical activity was assessed by ANOVA analysis. The Friedman’s post-hoc was also calculated.

All the analyses were carried out using STATISTICA (StatSoft, Inc. version 13, www.statsoft.com) and *P* < 0.05 was considered as statistically significant.

## Results

First of all, the reliability of the long version of the IPAQ was evaluated by determining the internal consistency. The internal consistency (coefficient Cronbach’s α) for the IPAQ was 0.62, which indicated a moderately acceptable reliability and internal consistency. The subscales of IPAQ Cronbach-Alpha reliability coefficient were observed to vary between 0.57 to 0.62.

Based on data collected from IPAQ-LV, it was established that almost 65% of the students represented a high level of physical activity. In this category physiotherapy students formed the largest group (72.3%). Whereas, the moderate activity category was most often represented by dietetics students (41.1%). Only 1.4% of all the surveyed students were classified as representing a low physical activity level (Table 1).

**Table 1:** Characteristics of physical activity levels and the time spend sitting according to the IPAQ-LV, among the students of different fields of health related faculty

Category	All students (*n* = 216)	MET-minutes/ week	Physiotherapy students (*n* = 101)	MET-minutes/ week	Dietetics students (*n* = 42)	MET-minutes/ week	Pharmacy students (n = 73)	MET-minutes/ week
	%	Mean (±SD)	%	Mean (±SD)	%	Mean (±SD)	%	Mean (±SD)
Low PA	1.4	453.7 ± 48.3	1.0	420.0 ± 0.0	0.0	0.0	2.7	470.5 ± 54.4
Moderate PA	33.8	1852.1 ± 665.1	26.7	2017.7 ± 691.1	38.1	1879.2 ± 710.5	41.1	1688.7 ± 596.0
High PA	64.8	8692.6 ± 7554.2	72.3	10,448.4 ± 9341.8	61.9	6805.6 ± 5006.9	56.2	6763.0 ± 3714.0
Total sitting		2781.8 ± 1238.5		2797.4 ± 1307.0		2215.7 ± 1230.1		3086.0 ± 1032.1

(*PA* Physical activity)

Taking into account the time spent sitting it turned out that all students spent this way on average 2781.8 ± 1238.5 MET-minutes/week. A detailed analysis of particular fields of health related faculty revealed that the pharmacy students spent on average more than seven hours a day sitting (3086.0 ± 1032.1 MET-minutes/week), physiotherapy students - almost seven (2797.4 ± 1307.0 MET-minutes/week), while the dietetics students about five hours a day (2215.7 ± 1230.1 MET-minutes/week) (Table 1).

The comparison of the students of different fields of health related faculty in terms of their time spent sitting showed statistically significant differences between dietetics and pharmacy students (*d* = 0.766; *g* = 0.785; *P* = 0.001), as well as dietetics and physiotherapy students (*d* = 0.458; *g* = 0.453; *P* = 0.015). In both cases, the dietetics students spent the least time sitting.

**Fig. 1:**
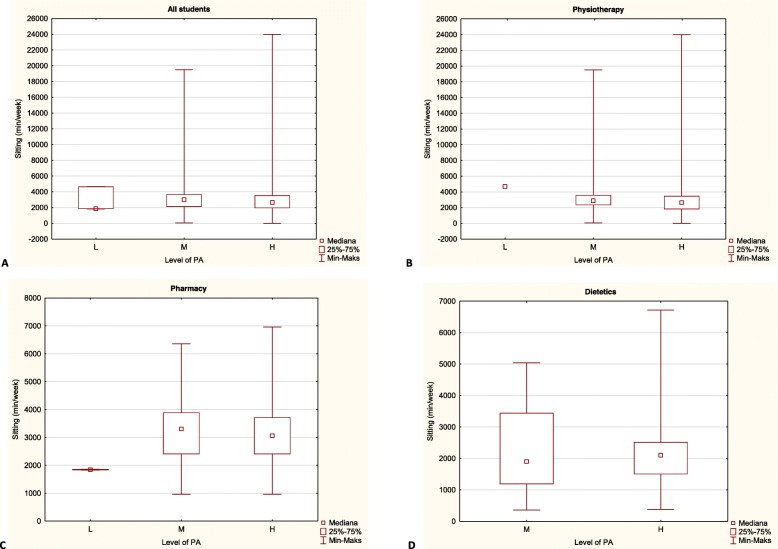
The comparison of the time spent sitting and physical activity level among all students and students of particular fields of health related faculty. (*L* Low, *M* Moderate, *H* High)

When assessing the dependency between the time that students spent sitting with their level of physical activity according to IPAQ-LV, it was observed that despite the declared many hours of sedentary behaviour, the vast majority of students were highly physically active. Statistical analysis showed no significant differences (*P* = 0.6880) between the time spent sitting and level of physical activity among all students. A detailed analysis of this relationships among students of particular fields of health related faculty also did not reveal statistically significant differences (*P* > 0.05). The characteristics of the time spent sitting and the level of physical activity among all students and students of particular fields of health related faculty was presented in the Fig. 1.

## Discussion

The beneficial effects of physical activity on health are emphasized more and more often. At the same time, it is also mentioned that insufficient physical activity or sedentary lifestyle may lead to the development of many diseases and increase general mortality. In this context, it seems important to conduct research that will determine the level of activity and time spent sitting in different groups and populations. One of the groups whose lifestyle should be monitored are students of medical universities. The relation between physical activity and the time spent sitting has an impact not only on their health, but indirectly it also contributes to the promotion of specific healthy habits among patients with whom they will work in the future. For this reason, in our study we focused primarily on the estimation of the time students spend sitting. It was found that on average they spend more than 46 h a week (2781.8 ± 1238.5 MET-minutes/week) and almost seven hours a day in a sitting position. These results are similar to those obtained in the other studies. For example, Papathanasiou et al. noticed that students spent more than seven hours a day (about 50 h a week) sitting [19], while Loginov et al. observed that students of general universities spent about 50 h a week sitting and those studying at technical universities about 44 h [20]. Another study assessing physical activity and sedentary time among students, however, conducting with using accelerometers not IPAQ, also showed that they spent on average 458.6 min/day being involved in sedentary activities, which corresponded to almost eight hours a day and 54 h a week [15].

The collected data enabled us to make a comparison between the level of physical activity and time spent sitting among the health related faculty students. Most of the students were highly or moderately physically active regardless of how much time they spent sitting. Rosenberg et al. showed that the participants who were sitting a lot were classified as representing physical activity categories just like those who were sitting the least [6]. In turn, Bauman et al. observed that as the time spent sitting became longer, the low level of physical activity increased whereas the high level decreased [1].

Based on the above-mentioned research it can be said that time spent sitting is not associated with physical activity. That is why it can-not be directly interpreted that people who spend many hours sitting are inactive. Much more often they follow current recommendations for physical activity and can be classified as moderately or even highly active people. For example, students who spend most of the day learning in a sitting position can also go to the gym or choose swimming or cycling for a couple of hours during the week. This way they are simultaneously sedentary and physically active people.

A major strength of this study is that we focused on health related faculty students, because this group of students has so far been rarely analyzed. We chose students of Physiotherapy, Pharmacy and Dietetics because in their future work they will spend many hours with their patients, during which they establish a close relationship, thanks to which they often become a role model and can transfer important knowledge about an active lifestyle.

Our study has some limitations. First of all, the number of students in particular fields of health related faculty were not the same, which could have influenced the results. However, this is due to the number of students in individual fields of study. Some fields are very numerous, while others are not.

Secondly, we used the IPAQ and self-report surveys, which may result in over- or underestimation of the results. On the other hand, the IPAQ is a widely used tool for assessment of physical activity and time spent sitting. It can be used in large study populations since it is inexpensive, simple and non-invasive [6, 18, 19, 21, 22]. Its validity and reliability have been proven in numerous studies [6, 23–25]. Moreover, to avoid the problem of misreporting due to a lack of understanding of the IPAQ questions [22], we introduced an interviewer to the study, so our results can be compared with other international studies.

Future research on sedentary behaviours should be conducted on large, standardized groups using objective measurement methods. It is worth assessing the time spent sitting in different areas of life [1, 6] as well as the types of sedentary activities to which people devote the most time because not all of them are equally harmful to health [4]. This will allow for supplementing healthy lifestyle recommendations with information on time spent sitting. It may be useful for many people to determine the minimum time spent sitting that is safe for health [1, 3]. Research has shown that modification of sedentary behaviours may bring more promising results in terms of promoting a healthy lifestyle than increasing a physical activity level [5].

## Conclusions

Based on the obtained results, many hours spent sitting should not be associated with physical inactivity. Most of the students of health related faculty perform physical activity but at the same time spend too much time in sitting position. Therefore, different university programmes should be develop to give more opportunities for students to be more active during their university classes. Moreover, while monitoring sedentary behaviour it would be recommended to complete it by physical activity assessment.

## Data Availability

All data generated or analysed during this study are included in this published article.

## Abbreviations

H: High
IPAQ/IPAQ-LV: the International Physical Activity Questionnaire/ the International Physical Activity Questionnaire-long version
L: low
M: moderate
MET: the metabolic equivalent of task
PA: the physical activity
vs: versus
